# Prevalence of Corneal Astigmatism in Patients before Cataract Surgery in Northern China

**DOI:** 10.1155/2014/536412

**Published:** 2014-06-03

**Authors:** Xiaoyong Yuan, Hui Song, Gang Peng, Xia Hua, Xin Tang

**Affiliations:** ^1^Tianjin Eye Hospital, Tianjin Key Laboratory of Ophthalmology and Vision Science, Clinical College of Ophthalmology, Tianjin Medical University, No. 4 Gansu Road, Heping District, Tianjin 300020, China; ^2^Department of Bioinformatics and Computational Biology, University of Texas, MD Anderson Cancer Center, Houston, TX 77030, USA

## Abstract

*Purpose*. To analyze the prevalence and presentation patterns of corneal astigmatism in cataract surgery candidates in a teaching hospital in northern China. *Methods*. From May 1, 2012, to April 30, 2013, partial coherence interferometry (IOLMaster) measurements of all qualified cataract surgery candidates were retrospectively collected and analyzed. *Results*. The study evaluated 12,449 eyes from 6,908 patients with a mean age of 69.80 ± 11.15 (SD) years. The corneal astigmatism was 0.5 diopters (D) or less in 20.76% of eyes, 1.0 D or more in 47.27% of eyes, 2.0 D or more in 13.16% of eyes, and 3.0 D or more in 3.75% of eyes. With-the-rule astigmatism was found in 30.36% of eyes, while against-the-rule was found in 52.41% of eyes. The percentage of against-the-rule astigmatism increased with age. *Conclusion*. Our study showed that almost one-half of preoperative eyes (47.27%) in northern China have a corneal astigmatism of 1.0 D or more, indicating that more surgical techniques or toric IOLs are needed to achieve better visual rehabilitation.

## 1. Introduction

Phacoemulsification is one of the most successfully and commonly performed cataract surgeries worldwide. With the development of modern surgical techniques and intraocular lenses (IOLs), patients expect and demand refractive error correction after cataract surgery. Advances in the calculation of IOL power have significantly reduced the incidence of spherical refractive errors, while residual astigmatism after surgery is a concern for both ophthalmologists and patients and can leave patients with symptomatic decreased visual function [1–5].

Partial coherence interferometry (IOLMaster, Carl Zeiss Meditec, Berlin, Germany) is widely used due to its superior performance in the measurement of ocular axial length. The IOLMaster analyzes six light reflections projected onto the anterior corneal surface within a 2.3 mm radius and can also be used as an automated keratometer (AK). Recent reports have shown that IOLMaster can precisely measure preoperative corneal astigmatism and can predict the residual corneal astigmatism after cataract surgery [6].

The distribution and prevalence of corneal astigmatism in cataract patients of different countries have been previously reported [5, 7–10]. An estimated 13,780,000 cases of blindness have been caused by cataracts in China [11] and two groups from Guangzhou [8] and Shanghai [12] have reported the distribution of corneal astigmatism before cataract surgery in southern and central China. However, there are no similar reports for cataract patients in northern China. This study reviewed all of the cataract cases in one year in one of the largest eye hospitals in China to investigate the prevalence of corneal astigmatism in a large sample in northern China. The findings may aid hospitals and manufacturing companies in evaluating the requirement for the use of toric IOLs or other reported surgical methods.

## 2. Patients and Methods

Retrospective biometry data were collected for all patients who had routine cataract surgery at the Tianjin Eye Hospital between May 1, 2012, and April 30, 2013. Cataract patients with a history of ocular surgery, corneal disease, and inflammation and with an age younger than 30 years old and a dense cataract that did not allow IOLMaster measurement were excluded. Routine eye examinations were performed before operation, including visual acuity, refraction, tonometry, slit lamp evaluation, and dilated fundus examination. The study was approved by the Human Research Ethics Committee at the Tianjin Eye Hospital and all procedures adhered to the tenets of the Declaration of Helsinki. All patients provided written informed consent. Three experienced technicians collected the keratometric data for consecutive patients using IOLMaster version 5.3 and the mean of five measurements was used for the parameters.

Data were analyzed by the R software package version 2.15.2 R Core Team (R Foundation for Statistical Computing, Vienna, Austria, ISBN 3-900051-07-0, URL http://www.R-project.org/). The Kolmogorov-Smirnov test was used to evaluate the normal distribution of variables. The results showed that the data were normally distributed, except for the data regarding astigmatisms. One-way analysis of variance and the Kruskal-Wallis test were applied for the comparison of variance for normally and nonnormally distributed data among different age groups, respectively. The *t*-test was used to compare keratometry between the two groups and a Wilcoxon signed rank test was used to compare corneal astigmatism data. Spearman's rank test was used to assess the relationship between age and astigmatism. A *P* value less than 0.05 was considered statistically significant.

## 3. Results

**Figure 1:**
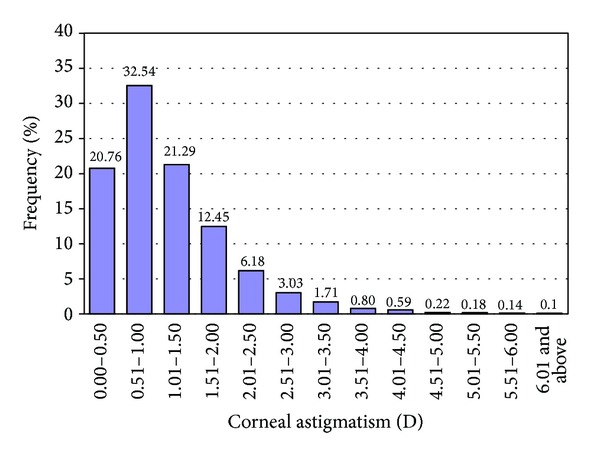
Distribution of corneal astigmatism in 0.5 D increments for all 12,449 eyes.

This study was composed of 12,449 eyes from 6,908 patients. The patient demographics are shown in Table 1, which also shows a comparison of 5 other published papers.  Figure 1 presents a histogram of the frequency distribution of corneal astigmatism. Among all of the patients, astigmatism of 0.51 to 1.00 D was the most common cylinder value (32.54%), followed by 1.01 to 1.50 D (21.29%) and 0.0 to 0.50 D (20.76%). In total, 3200 eyes (25.41%) exhibited a corneal astigmatism of 1.5 D or greater.

Table 2 presents the descriptive flat keratometry (K1) and steep keratometry (K2) in the 7 age groups. A gradually increasing keratometry value was observed with age, particularly in K2. Most eyes in this cohort were between 71 and 80 years old, which represented more than one-third (36.28%) of all cases. Patients between 61 and 70 years old represented one-fourth (25.91%) of all cases.

**Figure 2:**
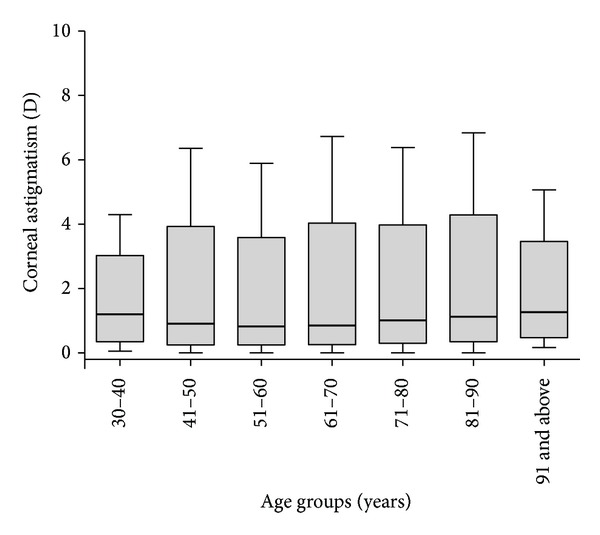
Corneal cylinder in all 7 age groups. The bold lines in the boxes represent the median (50% percentile), the upper and lower limits of the box represent the first quartile (25% percentile) and third quartile (75% percentile), and the bars represent the minimum and maximum values.

**Figure 3:**
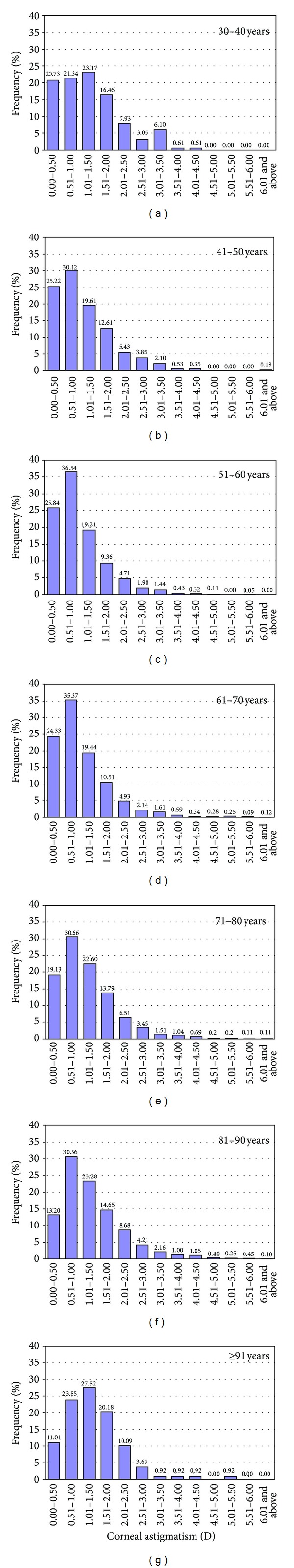
Frequency distribution of corneal astigmatism in 0.50 D steps for the 7 age groups.

Figure 2 shows the corneal astigmatism values in each age group. Spearman's rank correlation between age and astigmatism was *r* = 0.12 with *P* < 0.001.  Figure 3 depicts the distributions of corneal astigmatism in the different age groups.

**Figure 4:**
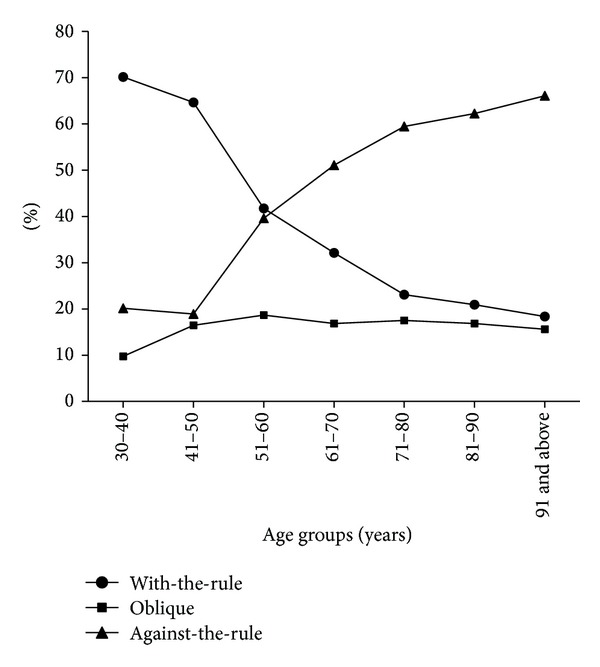
Percentages of WTR, ATR, and oblique corneal astigmatisms in the 7 groups.

With-the-rule (WTR, the steep meridian of the cornea being within 90 ± 30 degrees) corneal astigmatism was found in 3779 eyes (30.36%), against-the-rule (ATR, the steep meridian of the cornea being within 180 ± 30 degrees) corneal astigmatism was found in 6524 eyes (52.41%), and oblique (neither WTR nor ATR) corneal astigmatism was found in 2146 eyes (17.22%). The ATR astigmatism proportion increased with age and the WTR astigmatism proportion decreased with age, except in the 30–40-year-old age group, which showed a slightly higher percentage of ATR astigmatism (Figure 4).

No significant difference was found between the right and left eyes in K1 (43.91 ± 1.76 versus 43.94 ± 1.66, *t* = 0.75, *P* = 0.45) or K2 (45.08 ± 1.73 versus 45.06 ± 1.72, *t* = 0.66, *P* = 0.51). A statistically significant difference was found between right and left eye corneal astigmatisms with the Wilcoxon signed rank test (1.17 ± 0.85 D versus 1.13 ± 0.82 D, statistic = 19840457, *P* = 0.02).

The K1 and K2 values in females were higher than those in males (K1: 44.11 ± 1.67 versus 43.71 ± 1.74, *t* = 13.20, *P* < 0.0001; K2: 45.30 ± 1.72 versus 44.82 ± 1.80, *t* = 15.34, *P* < 0.0001). The corneal astigmatism in females was significantly greater than that in males according to the Wilcoxon signed rank test (1.19 ± 0.87 versus. 1.11 ± 0.80, statistic = 18500144, *P* < 0.0001).

## 4. Discussion

This study determined the distribution of corneal astigmatism in different age groups in northern China. Several studies have investigated the prevalence of corneal astigmatism using IOLMaster [5, 7, 9, 12–14], which not only affords the measurement of corneal status but also enables the easy and reliable calculation of IOLs as well as postoperative refraction data. The IOLMaster database was accessed for all cataract candidates in an entire year. The results showed that the mean age was slightly younger than previously reported data [8, 12] and that the 71–80-year-old age group occupied 36.28% of all cases, followed by the 61–70-year-old age group (25.91%) and the 81–90-year-old age group (16.01%); these results were similar to those of Chen et al. report from Guangzhou [8] but differed from those of Khan and Muhtaseb's report [5]. Khan et al. reported that the 71–80-year-old age group was the largest, followed by the 81–90- and 61–70-year-old age groups [5]. In terms of gender distribution, our study showed that the number of female patients was greater than that of males, which is consistent with other published studies [5, 7, 8, 12].

ATR astigmatism was the predominant group, comprising 52.41% of the cases, and the prevalence increased with age, except for the 30–40-year-old age group, which showed a slightly higher percentage than the 41–50-year-old age group. Selection bias may account for this finding because the 30–40-year-old age group represented only 1.32% of all cases. By contrast, the percentage of WTR astigmatism decreased with age. These findings are generally consistent with those of previous studies [7, 13, 15, 16].

The mean corneal astigmatism of this cohort was 1.15 ± 0.84 D (range from 0.0 to 6.84 D), which is slightly higher than that in other published studies [5, 7, 8, 12, 15]. The right (1.17 D) and left eyes (1.13 D) significantly differed, which is in contrast to the findings of Hoffmann and Hütz's study [15]. Interestingly, the corneal astigmatism in females (1.19 D) was significantly greater than that in males (1.11 D).

In our study, 20.76% of eyes had a corneal astigmatism of 0.5 D or less, which is lower than the results from other groups [5, 7, 8, 12]. A large proportion of eyes (47.27%) had a corneal astigmatism of 1.0 D or greater, which is higher than the results reported by the abovementioned groups. Additionally, 3.75% of eyes had 3.0 D of corneal astigmatism [8, 12], which is a greater prevalence than that reported by the other two Chinese studies (2.22%, 3.52%) but is lower than that reported by European studies (5.61%, 4.61%) [5, 7]. All age groups showed a similar distribution pattern of corneal astigmatism, except for the 30–40- and above 91-year-old age groups, which showed some variation in the astigmatism distribution. A previous study showed a similar distribution pattern [5, 7].

Several techniques exist to correct corneal astigmatism, including limbal relaxing incisions [17], opposite clear corneal incisions [18], excimer laser refractive procedures [19, 20], femtosecond laser-assisted astigmatic keratotomy [21, 22], and toric intraocular lens (IOL) implantation [23–25]. The procedure chosen primarily depends on the precise measurements of preoperative corneal astigmatism.

A clear corneal incision may result in a surgically induced corneal astigmatism in patients with 0.5 D [26]. In our study, 53.30% of eyes had a corneal astigmatism of 1.0 D or less and received sufficient correction through the performance of on-axis phacoemulsification combined with monofocal IOL implantation [5]. Meanwhile, 23.41% of eyes exhibited more than 1.50 D of corneal astigmatism in our study, which is similar to the findings of Khan et al. (20.5%) [5] and Ferrer-Blasco et al. (22%) [7], although their studies required more manipulations or techniques to correct for better visual rehabilitation. Other techniques, such as manual or femtosecond laser-assisted arcuate keratotomy, were used to correct much worse corneal astigmatisms [27, 28]. Recently, limbal femtosecond laser-assisted intrastromal arcuate keratotomy has been used for corneal astigmatisms of 1.50 ± 0.47 D [22]. However, the results of this procedure are unpredictable or are associated with complications [29, 30]. Considering the high cost of femtosecond laser surgery, the majority of the population in China cannot afford such procedures.

Toric IOLs have been used clinically since they were first described by Shimizu et al. [31], with encouraging results [32–35]. An analysis of the distribution of corneal astigmatism in a large cohort of cataract candidates will provide valuable information and benefits for manufacturers, ophthalmologists, and cataract patients. At present, toric IOLs can be used to correct corneal astigmatisms from 0.4 D to 8.4 D [7] during cataract surgery. In our case series, 1.51 D to 3.50 D represented 23.37% of all cases, most of which could be effectively corrected with toric IOLs. The higher cost of new IOLs may be another burden for patients and health insurance companies.

Corneal astigmatism changes significantly with age [15, 36–39]. Our study and the two previous Chinese investigations support this tendency [8, 12]. The mean values of K1, K2, corneal astigmatism, and other parameters in our study were slightly higher than those reported by some other studies [5, 7] but closely resemble those from reports from Shanghai [12] and Guangzhou [8], China. Possible reasons may be the inclusion of different racial and ethnic groups, different inclusion criteria, and different age distributions, among others. Our retrospective study was clinically based, which may lead to some selection bias. One advantage of our study is that we selected all of the cataract surgery candidates from an entire year, which presented more than one-half of cataract surgery cases in the same year in Tianjin, a city with 12,280,000 people http://en.wikipedia.org/wiki/Tianjin.

In conclusion, our study revealed the distribution of all cataract candidates in one year in a single hospital in northern China. A number of our cases (47.27%) exhibited a corneal astigmatism of 1.0 D or more. Corneal astigmatism increases with age. With an aging population and a higher demand for improved vision, the need for astigmatism correction with toric IOLs or other methods will increase accordingly.

## Figures and Tables

**Table 1:** Patient demographics compared with 5 other published studies.

	Present	Guan et al. [12]	Chen et al. [8]	Ferrer-Blasco et al. [7]	Khan and Muhtaseb [5]	De Bernardo et al. [14]
Eyes/patients	12449/6908	1430/827	4831/2849	4540/2415	1230/746	757/380
Age (y)						
Mean ± SD	69.80 ± 11.15	72.27 ± 11.59	70.56 ± 9.55	60.59 ± 9.87	75.54 ± 0.71	71.89 ± 10.19
Range	30, 97	16, 98	49, 95	32, 87	30, 104	33, 96
Male/female	3199/3709	359/468	1090/1759	768/1647	343/403	176/204
Corneal astigmatism (D)						
Mean ± SD	1.15 ± 0.84	1.07 ± 0.73	1.01 ± 0.69	0.90 ± 0.93	1.03 ± 0.73	1.02 ± 0.69
Range	0.0, 6.63	0.06, 5.52	0.05, 6.59	0.25, 6.75	0.0, 6.2	0.06, 4.57
K1 mean ± SD	43.93 ± 1.67	43.57 ± 1.56	43.76 ± 1.53	43.48 ± 1.61	43.43 ± 1.49	43.54 ± 1.43
K2 mean ± SD	45.08 ± 1.73	44.64 ± 1.65	44.76 ± 1.56	44.08 ± 1.59	44.46 ± 1.56	44.56 ± 1.52
Corneal astigmatism (%)						
≤0.5 D	20.76	21.2*	23.14	58.8	24.47	23.38
≥1.0 D	47.27	45.37	41.3	34.8	40.4	41.74
≥2.0 D	13.16	10.33	8.22	9.26**	9.67	8.32
≥3.0 D	3.75	2.22	3.52	5.61***	4.61	2.64

*Not including 0.5 D, **not including 2.0 D, and ***not including 3.0 D.

D = diopter, K1 = flat keratometry, and K2 = steep keratometry.

**Table 2:** Descriptive statistics by age group.

Age group (y)	Astigmatism (D)	K1 (D) mean ± SD	K2 (D) mean ± SD	Eyes (%)
30–40	1.33 ± 0.85	42.77 ± 2.23	44.10 ± 2.38	164 (1.32)
41–50	1.10 ± 1.10	43.51 ± 1.83	44.61 ± 1.92	571 (4.59)
51–60	0.99 ± 0.71	43.91 ± 1.61	44.90 ± 1.70	1869 (15.01)
61–70	1.05 ± 0.80	44.04 ± 1.63	45.10 ± 1.70	3226 (25.91)
71–80	1.20 ± 0.83	43.95 ± 1.65	45.14 ± 1.69	4517 (36.28)
81–90	1.34 ± 0.90	43.95 ± 1.68	45.28 ± 1.70	1993 (16.01)
≥91	1.39 ± 0.82	43.73 ± 1.65	45.12 ± 1.76	109 (0.88)
P*	<0.001	<0.001	<0.001	<0.001

D = diopter, K1 = flat keratometry, and K2 = steep keratometry.

*Kruskal-Wallis test.
